# A comparison between wireless CROS/BiCROS and soft-band BAHA for patients with unilateral hearing loss

**DOI:** 10.1371/journal.pone.0212503

**Published:** 2019-02-21

**Authors:** Ji Eun Choi, Sun Mi Ma, Heesung Park, Yang-Sun Cho, Sung Hwa Hong, Il Joon Moon

**Affiliations:** 1 Department of Otorhinolaryngology - Head and Neck Surgery, Dankook University Hospital, Cheonan, Republic of Korea; 2 Hearing Research Laboratory, Samsung Medical Center, Seoul, Republic of Korea; 3 Department of Otorhinolaryngology - Head and Neck Surgery, Samsung Medical Center, Sungkyunkwan University School of Medicine, Seoul, Republic of Korea; 4 Department of Otorhinolaryngology - Head and Neck Surgery, Samsung Changwon Hospital, Sungkyunkwan University School of Medicine, Seoul, Republic of Korea; Medical University Hannover; Cluster of Excellence Hearing4all, GERMANY

## Abstract

This study directly compared the performance of a contralateral routing of signal (CROS)/bilateral routing of signal (BiCROS) and a soft-band bone-anchored hearing aid (BAHA) in patients with unilateral sensorineural hearing loss (SNHL) and assessed the relationship between hearing aid benefits and personal factors. Participants with unilateral SNHL were prospectively enrolled in the study and were tested under the following three conditions: unaided, with CROS/BiCROS, and with soft-band BAHA. Sound localization, consonant, hearing in noise, and psychoacoustic tests were performed. Pseudobinaural benefits (e.g., squelch, summation, and head shadow effect) were obtained in both the CROS/BiCROS and soft-band BAHA conditions and compared to the unaided condition. Sound localization ability was not improved in either the CROS/BiCROS condition or soft-band BAHA condition. Rather, sound localization ability was significantly decreased in the CROS/BiCROS setting. A CROS/BiCROS hearing aid and a soft-band BAHA provided additional benefit for speech-in-noise perception when target speech was directed to the impaired ear side. The CROS/BiCROS hearing aid was superior to the soft-band BAHA one in decreasing the head shadow effect, but it appeared to have a negative effect when the noise was delivered to the better ear. The positive and negative effects of CROS/BiCROS for localization and speech perception were significantly correlated with personal factors such as age, hearing threshold in the better ear, and unaided psychoacoustic performances. Despite the lack of device acclimatization, we believe that this study provides counseling information for hearing aid clinics to use in the context of patients with unilateral SNHL.

## Introduction

Individuals with severe to profound unilateral sensorineural hearing loss (SNHL) report difficulty localizing sound within a horizontal plane and with listening to sounds coming from the side of the impaired ear [1]. Despite having normal to near-normal hearing in one ear, these individuals experience difficulty with understanding speech, particularly in reverberant or noisy environments [2–4]. These difficulties occur due to the loss of binaural hearing and the head shadow effect [1]. In binaural hearing, the differences in timing and level cues for a sound arriving to the two ears are critical for processing complex auditory signals, such as speech perception in noisy conditions and the localization of sound. However, individuals with severe to profound unilateral SNHL lose these essential auditory cues because all sounds arrive to the nonimpaired ear at the same time and level. Also, the head shadow effect, where the head acts as a barrier to sound as it travels from the impaired ear side of the head to the functioning ear side of the head, makes it hard to hear sound coming from the direction of the impaired ear [5].

There are two main nonsurgical options currently used worldwide to help alleviate the head shadow effect. One option is a rerouting of the signal to the contralateral normal hearing ear through air conduction. This can be accomplished by use of the contralateral routing of signal (CROS) system. CROS/bilateral routing of signal (BiCROS) air conduction hearing aids use a microphone in the impaired ear that delivers sound to the functioning ear either via a wire or wirelessly. BiCROS hearing aids also provide appropriate amplification for the functioning ear when the better ear has hearing loss. The second option is the use of a nonsurgical bone conduction system (e.g., Baha Softband and SoundArc, Cochlear or the ADHEAR System, MED-EL). Bone-anchored hearing aids (BAHAs) route sound to the better ear by transcranial bone conduction from a microphone/processor located on the skull of the impaired ear side. A bone conduction audio processor coupled to the head with a soft band has been the most common nonsurgical option available over the past 15 years or so. Thus, wearing a CROS hearing aid or a soft-band BAHA on the impaired side limits the head shadow effect. Although both devices impair speech perception when the noise emanates from the impaired ear side [6–9] and do not improve sound localization abilities [6, 10–14], they do improve speech perception when noise is delivered to the better ear [9, 10, 12, 15–17].

Recently, more individuals with severe to profound unilateral SNHL consider their hearing loss to be a hindrance in social interactions, which affects their emotional and psychological well-being [18–20]. Patients and physicians alike are urged to recognize the detrimental effects of unilateral SNHL and to choose a rehabilitative option for unilateral SNHL. Because the difficulties caused by unilateral SNHL differ between individuals (likely due to contralateral hearing, types of social and professional activities, and coping strategies), the design of a tailored rehabilitation strategy for each individual is required. Previous studies comparing wired CROS hearing aids and BAHAs have generally concluded that the latter is preferred for unilateral SNHL, particularly because of the subjective scores achieved in the speech-in-noise perception tests [6, 10, 11, 14, 17]. Since these studies were conducted, however, both devices have been enhanced in different ways to improve sound quality, such as by increasing the sophistication of their signal processing and the adaptive directionality of the microphones. Therefore, a comparison of the performance of currently available wireless CROS/BiCROS hearing aids and soft-band BAHAs in patients with unilateral SNHL is needed. In addition, the effects of a CROS/BiCROS hearing aid or soft-band BAHA on psychoacoustic performance such as spectral and temporal resolution have not yet been determined to date.

The present study compared the performances of localization ability in a horizontal plane, pseudobinaural benefits of speech perception in noisy conditions, and psychoacoustic performance between wireless CROS/BiCROS hearing aids with technologies such as digital noise reduction and directional microphone and soft-band BAHAs with similar technologies. To provide a customized rehabilitation strategy, we also assessed the relationship between hearing aid benefit and personal factors.

## Methods

### Ethics statement

All participants were recruited and tested in the Hearing Laboratory at Samsung Medical Center in Seoul, South Korea. Every participant provided written informed consent prior to participating in this study. Written informed consent was additionally obtained from the next of kin, caretakers, or guardians of any minors/children enrolled in this survey. The study protocol was approved by the Samsung Medical Center Institutional Review Board (2016-06-136). The study was carried out in accordance with approved guidelines.

### Subjects

A total of 21 subjects (10 males and 11 females, age range: 15–71 years, mean age: 44 years) with unilateral SNHL were prospectively enrolled. All subjects had severe to profound hearing loss (threshold averages for 500, 1, 2, and 4 kHz > 70 dB HL) in one ear and normal hearing or mild hearing loss (threshold averages for 500, 1, 2, and 4 kHz < 40 dB HL) in the contralateral ear. None of the subjects had air-bone gaps of 15 dB or more at 500, 1, 2, or 4 kHz in both ears. Demographics and pure-tone thresholds for each subject are shown in Table 1.

**Table 1:** Demographics and pure-tone thresholds for each subject.

No.	Sex	Age	Tested ear	Pure-tone thresholds (air-conduction, dB HL)											
				Tested ear						Non-tested ear					
				250 Hz	500 Hz	1 kHz	2 kHz	4 kHz	8 kHz	250 Hz	500 Hz	1 kHz	2 kHz	4 kHz	8 kHz
S01	M	55	R	105	115	S/O	S/O	S/O	S/O	10	10	10	0	20	40
S02	M	31	L	105	115	110	105	105	105	20	25	25	25	40	40
S03	F	22	L	105	115	S/O	S/O	S/O	S/O	20	20	15	10	10	60
S04	F	65	R	65	60	65	95	110	100	30	10	25	50	50	50
S05	F	15	R	80	80	80	85	85	65	0	0	5	5	5	0
S06	M	67	L	80	75	80	75	60	65	10	5	15	20	50	50
S07	F	58	L	60	55	70	80	80	100	10	10	10	5	10	40
S08	F	18	L	S/O	S/O	S/O	S/O	S/O	S/O	0	0	0	0	10	10
S09	M	68	L	90	110	105	S/O	S/O	S/O	15	20	40	45	45	60
S10	F	60	R	S/O	S/O	S/O	S/O	S/O	S/O	10	15	20	35	50	50
S11	F	54	R	S/O	S/O	S/O	S/O	S/O	S/O	15	5	10	5	15	30
S12	F	42	L	100	105	S/O	S/O	S/O	S/O	45	30	25	20	60	55
S13	M	53	L	S/O	S/O	S/O	S/O	S/O	S/O	0	10	15	0	5	15
S14	M	20	L	90	100	90	90	95	S/O	15	10	10	5	5	5
S15	M	16	R	S/O	S/O	S/O	S/O	S/O	S/O	15	15	25	25	30	40
S16	F	22	L	55	65	80	70	90	100	10	5	5	5	15	5
S17	F	70	R	S/O	S/O	S/O	S/O	S/O	S/O	15	15	20	20	35	50
S18	M	36	L	95	105	115	S/O	S/O	S/O	15	10	35	0	5	5
S19	M	30	R	100	105	110	120	S/O	S/O	10	10	10	5	10	5
S20	M	71	L	95	90	115	120	75	85	20	20	25	40	55	60
S21	F	54	L	105	105	110	120	S/O	S/O	15	15	20	10	15	20

M: male, F: female, R: right, L: left, S/O: scale-out

### Hearing aids and fitting

The CROS/BiCROS system employed herein consisted of a wireless Phonak CROS transmitter on the poorer ear and a Phonak Audéo Q70-312T open-fit hearing aid (Phonak, LLC, Warrenville, IL, USA) on the better ear. If there was some degree of hearing loss in the better ear, a BiCROS hearing system was adapted. Devices were fitted by experienced audiologists using Phonak’s fitting algorithm (Adaptive Phonak Digital) as recommended by the manufacturer. The Phonak CROS/BiCROS program included (1) SoundFlow, a multi-base automatic program that actively adapts and integrates the correct parameters to ensure optimal everyday listening; (2) Rear Ear Sound, an adaptive directional microphone to improve sound localization and improve speech in noisy situations; and (3) Sound Recover, a frequency adjustment protocol that acts according to the threshold of the better ear. Detailed CROS/BiCROS hearing aid fitting information is shown in Table 2.

**Table 2:** CROS/BiCROS hearing aid fitting information.

Subject	Gain level	Dome type	Mode	Sound flow	Rear ear sound	Sound recover
S1	100	Power	CROS	On	Off	Off
S2	100	Closed	CROS	On	Off	Off
S3	100	Open	CROS	On	Off	Off
S4	100	Closed	CROS	On	Off	Off
S5	100	Closed	CROS	On	Off	Off
S6	100	Open	CROS	On	Off	Off
S7	100	Closed	CROS	On	Off	Off
S8	100	Closed	CROS	On	Off	Off
S9	100	Closed	CROS	On	Off	Off
S10	80	Open	BiCROS	On	On	On
S11	80	Open	BiCROS	On	On	On
S12	80	Closed	BiCROS	On	On	On
S13	80	Open	BiCROS	On	On	Off
S14	80	Closed	BiCROS	On	On	On
S15	80	Open	BiCROS	On	On	On
S16	80	Open	BiCROS	On	On	Off
S17	80	Open	BiCROS	On	On	On
S18	80	Open	BiCROS	On	On	Off
S19	80	Open	BiCROS	On	On	Off
S20	80	Closed	BiCROS	On	On	On
S21	80	Closed	BiCROS	On	On	On

The BAHA sound processor was worn on a BAHA Softband. The tested sound processor was the programmable Cochlear Baha 3 Power (BP110). The BAHA sound processor (with directional microphone) was fitted by experienced audiologists using the Baha Fitting Software. The Client, Indication, and Connection types were configured using the BC Select step. This enabled a quick setup of the sound processor and incorporated the latest research data on corrections for transcranial attenuation, cross-hearing, and transmission loss through the skin [21]. The final gain settings were established based on measurements of the actual thresholds through the BC Direct function of the fitting software [22]. The patients were not allowed to adjust the volume control setting during test procedures. None of the subjects had tried hearing devices such as CROS/BiCROS hearing aids, BAHAs, or conventional hearing aids prior to participating in this study.

### Test battery administration

Participant performance was compared under the following three different conditions: unaided, with the wireless CROS/BiCROS, and with the soft-band BAHA. Participants were randomly assigned to the CROS/BiCROS hearing aid or BAHA. The order in which hearing devices were worn varied across subjects. There was no acclimatization period for the devices. All subjects participated in audiogram, localization, speech perception, and psychoacoustic tests in a double-walled and sound-attenuating booth. In addition to objective measures, subjective performance was also assessed using a questionnaire. Each participant performed all tests on the same day. Sound localization and speech-in-noise perception tests were conducted without masking the better ear, whereas for audiogram, consonant test, and psychoacoustic tests, an ear plug was inserted in the better ear only in the BAHA condition.

### Sound localization

Each participant was seated one meter from the center of a 13-loudspeaker array. The loudspeakers were placed at 15° intervals from a −90° azimuth to a +90° azimuth. Study participants sat in chairs at the center of the arc with their ears at a height equal to the loudspeaker array. Participants were informed of the number corresponding to each speaker. They were instructed that the target word would be randomly presented from one of the 13 speakers. They were asked to remain seated, facing the loudspeaker at the 0° azimuth and to hold their head still during the testing. Each participant was instructed to say the number of the loudspeaker from which they thought the target sound was coming. The localization stimulus was a Korean bisyllabic word “ja-yeon.” The stimulus was 865 ms in duration. It was recorded from a male speaker using a sampling rate of 44.1 kHz [23]. Each stimulus was randomly presented at 65 dB SPL roved by ± 4 dB (61, 65, and 69 dB SPL) on each trial. For each subject, testing was conducted in the three different listening conditions: unaided, with the wireless CROS/BiCROS, and with the soft-band BAHA. The speech token was presented once from each of the 13 loudspeakers per block. The order of presentation was random across the 13-loudspeaker array. The process was repeated four times (4 blocks, 13 stimuli per block) per listening condition. Thus, a total of 12 blocks (4 blocks x 3 listening conditions) were performed for each participant. Prior to the experiment, two practice sets of serial presentations of every sound source direction and eight to 10 random presentations were administered. Feedback was provided during the practice, but not during the test situations.

Localization accuracy was quantified in two ways, as follows: root-mean square error (RMSE) and hemifield identification score. First, we calculated the error in degrees between the target speaker and the response speaker for each trial. Errors were squared before they were averaged. Then, the square root was taken after averaging (Eq 1). We also computed a hemifield identification score to reflect the accuracy of right/left discrimination. This score was computed by counting any response as correct when the speaker identified by the listener was located in the same half of the loudspeaker array as the target speaker. Therefore, the seventh loudspeaker (0°) was eliminated and lateralization responses were calculated for 12 loudspeakers sorted in two directions (six loudspeakers for each direction). Sound source locations were divided into two directions (better and worse ear sides). Results of hemifield identification scoring were converted to percent correct (%):

$$\text{RMSE}\;({}^{\circ})=\sqrt{\frac{\Sigma_{i=1}^{n}{\left(stimulus_{i}-response_{i}\right)}^{2}}{n}}$$ (1)

where n is the number of presented stimuli.

### Speech perception

Speech perception testing included the consonant test and the Korean Hearing in Noise Test (K-HINT) [24]. For the consonant test, study participants sat on a chair in the center of the sound field facing two loudspeakers located approximately one meter away at the 0° azimuth. The consonant test was performed in quiet, steady, and modulated noise (0 dB SNRs) situations. The target stimuli consisted of 18 Korean bisyllabic nonsense words (/a/-target consonant+/a/, i.e., /a-sa/ or /a-na/) that had been prerecorded with a male speaker. Target consonant and noise were presented at a calibrated level of 62 dB SPL from the front (0°). Each of the 18 target words was randomly presented three times. Participants were instructed to select the target word from the 18 words presented on the screen. The correct percentage (%) was calculated by dividing the number of correct answers by a total of 54 target words and multiplying by 100. Prior to the experiment, four to five practice tests were administered. No feedback was provided during the practice or test runs.

The participants were also subjected to K-HINT, which was modified for this experiment to measure the pseudobinaural benefit of speech perception in a noisy condition. During this trial, the study participant sat on a chair in the center of the sound field facing two loudspeakers that were located approximately one meter away at the 0° azimuth. Two additional speakers were located at the same distance from the listener at the ± 90° azimuths. Target sentences and white noise were presented from the front speaker (0°) or from one of the two side speakers (± 90°). The presentation level of the noise was fixed at 65 dBA, and the level of target sentences varied according to the adaptive procedure of the original HINT [25]. One list of 20 sentences was randomly selected from a total of 12 lists. Results of the modified K-HINT were presented as dB SNR; speech levels corresponded to 50% correct recognition of sentences in a noisy condition.

The K-HINT trial was conducted in three different conditions, as follows: (1) for measuring the summation effect, both speech and noise were presented from the front (F_S_F_N_); (2) for measuring the squelch effect, speech was presented from one of the two side speakers (± 90°) toward the better ear (NH), while noise was presented from one of the two side speakers (± 90°) toward the poorer ear (HL) (NH_S_HL_N_); and (3) for measuring the head shadow effect, speech was presented from one of the two side speakers (± 90°) toward the poorer ear, while noise was presented from one of the two side speakers (± 90°) toward the better ear (HL_S_NL_N_). Study participants were instructed not to move their heads. Prior to the experiment, four to five practice tests were administered. No feedback was provided during the practice or test runs.

### Psychoacoustic tests

A custom-made MATLAB (MathWorks, Natick, MA, USA) graphical user interface was used to present acoustic stimuli to subjects for psychoacoustic tests. Stimuli were presented through a loudspeaker (HS-50M; Yamaha Corp., Hamamatsu, Japan) in the sound field at an average level of 65 dBA. Study participants sat at a distance of one meter from the loudspeaker and were asked to face the speaker during the course of the experiment.

#### Temporal-modulation detection test

The temporal-modulation detection (TMD) test was performed using the method previously described by Won *et al*. (2011) [26]. For modulated stimuli, sinusoidal amplitude modulation was applied to a wideband noise carrier. The stimulus duration was one second for both modulated and unmodulated signals. For unmodulated stimuli, continuous wideband noise was applied. A modulation frequency of 10 Hz was tested. Modulated and unmodulated signals were gated on and off with 10-ms linear ramps and were concatenated with no gap between the two signals. The TMD threshold was measured using a two-interval, two-alternative adaptive forced choice paradigm. One of the intervals consisted of modulated noise and the other interval consisted of steady noise. Subjects were asked to identify the interval that contained the modulated noise. A two-down, one-up adaptive procedure was used to measure TMD threshold, starting with a modulation depth of 100% and decreasing in steps of 4 dB from the first to the fourth reversal and then 2 dB for the next 10 reversals thereafter. For each test run, the final 10 reversals were averaged to obtain the TMD threshold. TMD thresholds in dB relative to 100% modulation (i.e., 20log_10_ *m*_*i*_) were obtained, where m_*i*_ indicates the modulation index. Lower levels of modulation depth indicated better performance.

#### Spectral-ripple discrimination test

The spectral-ripple discrimination (SRD) test was administered using a previously established technique [27]. Three ripple noise tokens with a 30-dB peak-to-trough ratio, two with a standard ripple phase and one with an inverted ripple phase, were created using 2555 tones. The spectral modulation starting phase of the full-wave-rectified sinusoidal spectral envelope was set to zero radians for standard ripple stimuli. The spectral modulation starting phase of inverted spectral-ripple stimuli was set to π/2 radians. The bandwidth of the ripple spectrum was 100–5000 Hz. The duration of the stimuli was 500 ms.

The order of presentation of the three tokens was random, and the subject’s task was to select the “odd” stimulus. The stimuli were presented through a loudspeaker (HS-50M; Yamaha Corp., Hamamatsu, Japan) in the sound field at an average level of 65 dBA. To measure SRD thresholds, a three-interval, three-alternative forced choice paradigm with an adaptive two-up and one-down procedure was used. Ripple density varied between 0.125 ripples and 11.314 ripples per octave in equal ratio steps of 1.414 in an adaptive manner, with 13 reversals that converged to the 70.7% correct point [28]. A level attenuation of 1–8 dB (in 1-dB increments) was randomly selected for each interval in the three-interval task. The SRD threshold for each adaptive run was calculated as the mean of the last eight reversals. A higher spectral-ripple threshold indicated better discrimination performance.

### Questionnaires

For subjective performance of wireless CROS/BiCROS and soft-band BAHA, sound quality and annoying background noise were assessed using a six-item visual analog scale (VAS), where 0 represented being unable to hear and 5 indicated hearing perfectly. Device preference was also assessed. Participants were asked to select the preferred device between CROS/BiCROS and BAHA. Device preference was quantified on a 6-point scale with the rating of 0 indicating no device preference, 1 indicating very slightly better, 2 indicating slightly better, 3 indicating better, 4 indicating much better, and 5 indicating very much better. Positive and negative numbers were used to account for both directions of preference (negative for CROS/BiCROS and positive for BAHA). The resulting preference value was a mean rating score.

### Statistical analysis

Results were analyzed using SPSS 18.0 (SPSS Inc., Chicago, IL, USA). To compare the localization performance, word recognition, psychoacoustic performances, and subjective assessments between the listening conditions (unaided, with CROS/BiCROS, and with BAHA), repeated-measures one-way analysis of variance (ANOVA) or the Friedman test were conducted depending on the test of normality. If there were significant differences among the three listening conditions, post-hoc analysis with Bonferroni correction or the Wilcoxon signed-rank test was performed to evaluate differences between two different listening conditions (i.e., unaided vs. aided with CROS/BiCROS, unaided vs. aided with BAHA, and aided with CROS/BiCROS vs. aided with BAHA). Post-hoc analysis with Wilcoxon signed-rank test was conducted with a Bonferroni correction applied, resulting in a significance level of p < 0.017. A paired t-test was conducted to compare the hemifield identification between the better ear side and the poorer ear side in each listening condition.

Relationships between hearing aid benefit and personal factors (e.g., age, pure-tone thresholds in the better ear, and unaided TMD and SRD thresholds) were assessed using Pearson’s linear correlation coefficient. For these analyses, hearing aid benefit was calculated as the difference between aided and unaided conditions. The pure-tone thresholds averaged at 0.5, 1, 2, and 4 kHz in the better ear were used.

## Results

### Sound localization

**Fig 1:**
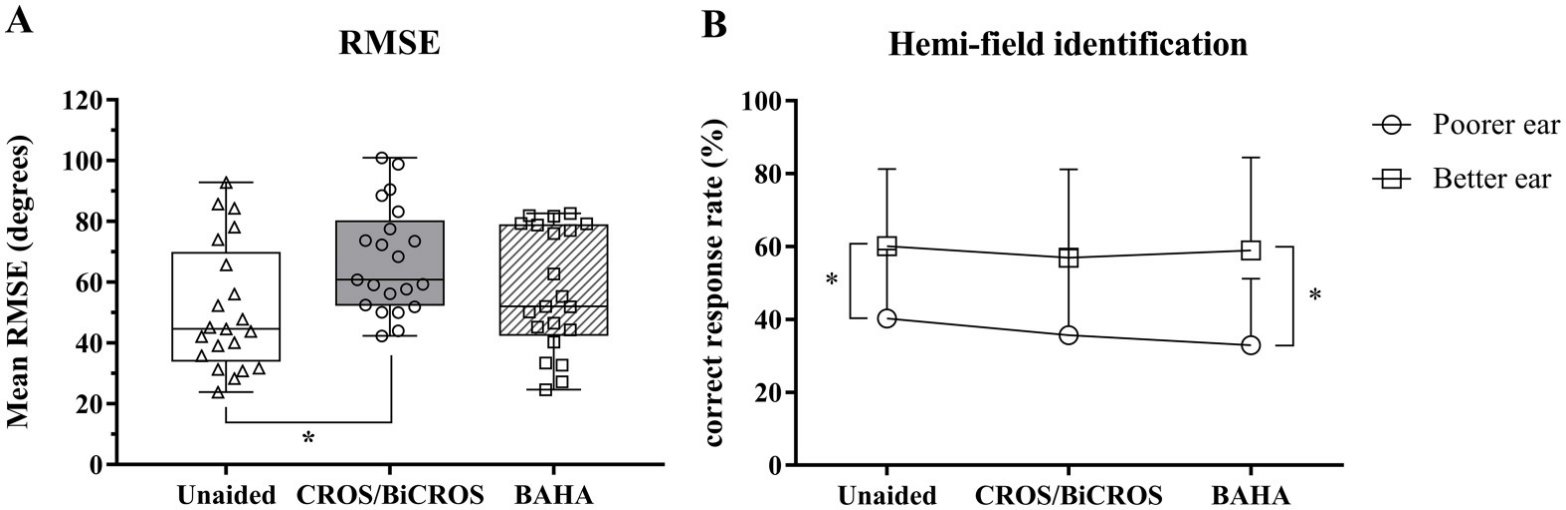
Localization results. Panel A shows the mean RMSEs for the three listening groups (unaided, aided with CROS/BiCROS, and aided with BAHA). Panel B shows the hemifield identification scores of the three listening groups. The hemifield identification score was calculated by counting the correct responses when the speaker identified by the listener was in the correct half of the loudspeaker array. Loudspeaker location was categorized as either on the poorer ear side or the better ear side. Error bars indicate SD. Asterisks (*) indicate significant differences, *p* < 0.05.

**Fig 2:**
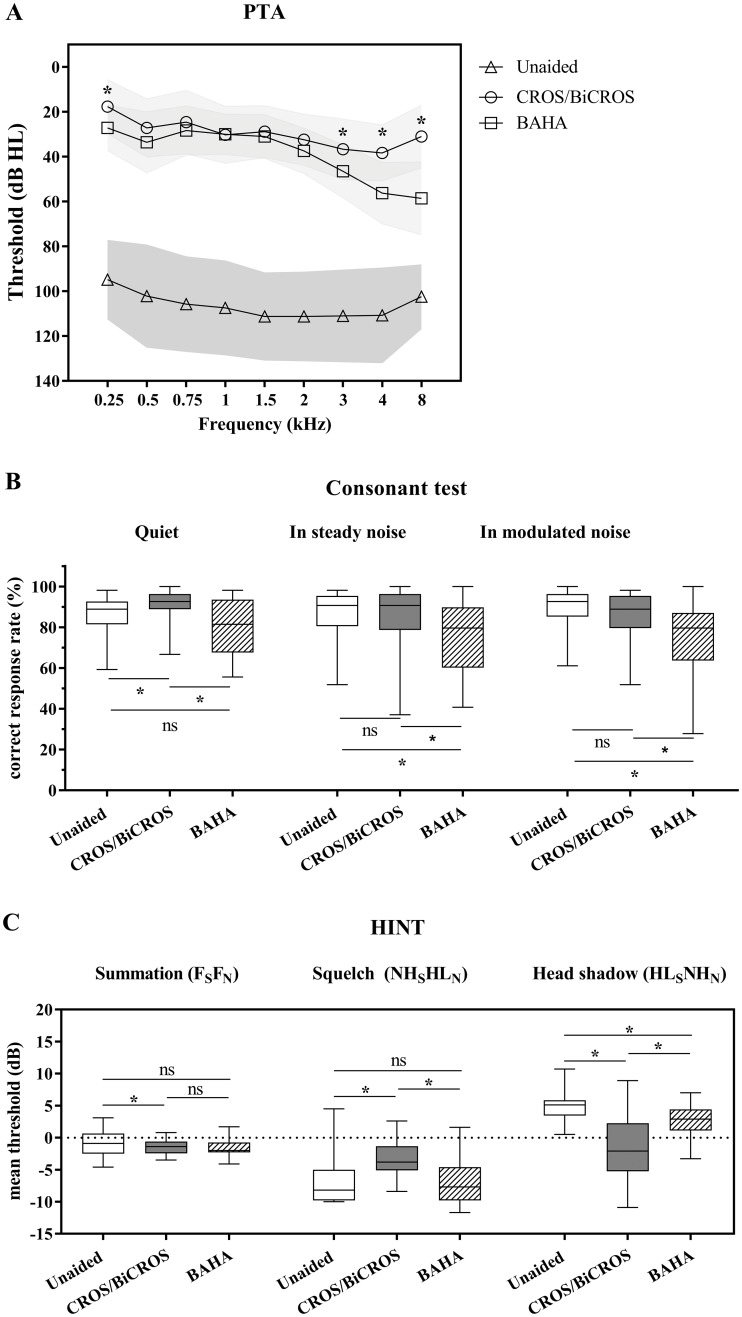
Speech perception. Panel A shows the aided pure-tone threshold at each frequency for the three listening conditions (unaided, aided with CROS/BiCROS, and aided with BAHA). If the pure-tone threshold is scaled out, it is expressed as 110 dB HL (for 250 Hz and 8 kHz) and 125 dB HL (for the rest of the frequencies). Panel B shows consonant perception in quiet and noisy backgrounds for the three listening conditions. Mean and SD are presented as box-and-whisker plots. Panel C shows pseudobinaural benefits of speech perception in noise for the three listening conditions. The SNR threshold was acquired in the presence of noise for the following three conditions: (1) for measuring the summation effect, both speech and noise were presented from the front (F_S_F_N_); (2) for measuring the squelch effect, speech was presented from one of the two side speakers (± 90°) toward the better ear (NH), while noise was presented from one of the two side speakers (± 90°) toward the poorer ear (HL) (NH_S_HL_N_); and (3), for measuring the head shadow effect, speech was presented from one of the two side speakers (± 90°) toward the poorer ear, while noise was presented from one of the two side speakers (± 90°) toward the better ear (HL_S_NL_N_). Asterisks (*) mean a significant difference; *p* < 0.05 in post-hoc tests using the Bonferroni correction.

The results of localization testing for participants with unilateral SNHL in each listening condition are shown in Fig 1. The mean ± standard deviation (SD) of the RMSEs in the three listening conditions (unaided, aided with CROS/BiCROS, and aided with BAHA) are presented as box-and-whisker plots (51.2 ± 20.9° for the unaided condition, 67.3 ± 17.6° for the CROS/BiCROS condition, and 57.3 ± 20.0° for the BAHA condition in Fig 2A). Repeated-measures ANOVA revealed that mean RMSE differed significantly between listening conditions [F(2, 40) = 7.739, *p* < 0.001]. Post-hoc tests using the Bonferroni correction revealed that mean RMSE was significantly increased when tested in the CROS/BiCROS condition as compared with in the unaided condition (*p* = 0.003). However, no significant difference in mean RMSE was observed when performance in the BAHA condition was compared to that in the unaided condition (*p* = 0.225). Separately, the mean ± SD values of hemifield identification score in the three listening conditions are presented according to the loudspeaker location (Fig 2B). Loudspeaker locations were divided into two directions (poorer ear side and better ear side). A repeated-measures ANOVA revealed that there was no significant difference in mean hemifield identification score between listening conditions for the poorer ear side [F(2, 40) = 1.687, *p* = 0.198] and for the better ear side [F(2, 40) = 0.335, *p* = 0.717]. A paired t-test was run for each listening condition to determine whether or not there was a statistically significant mean difference between the poorer ear side and the better ear side. Participants with unaided and BAHA conditions performed significantly better at differentiating the sound location side when the sound came from the better ear side versus when the sound came from the poorer ear side [difference of 19.8%, *t*(20) = 2.344, *p* = 0.03 for the unaided condition and difference of 26.0%, *t*(20) = 2.819, *p* = 0.011 for the BAHA condition]. There was no significant difference in mean hemifield identification score in the CROS/BiCROS condition, regardless of where the sound originated from [difference of 21.8%, *t*(20) = 2.063, *p* = 0.052].

### Speech perception

The mean ± SD of pure-tone thresholds between listening conditions are shown in Fig 2A. There were significant differences in pure-tone thresholds depending on listening condition (*χ*^*2*^(2) = 31.976–38.456, all *p* < 0.001 for all frequencies). Post-hoc analysis with Wilcoxon signed-rank test was conducted with a Bonferroni correction applied, resulting in a significance level set at *p* < 0.017. At 250 Hz, BAHA performed significantly better than did CROS/BiCROS (Z = −3.197, *p* = 0.001). At higher frequencies (3, 4, and 8 kHz), CROS/BiCROS performed significantly better than BAHA (Z = −3.178, *p* = 0.001 for 3 kHz; Z = −3.882, *p* < 0.001 for 4 kHz; and Z = −3.994, *p* < 0.001 for 8 kHz). Speech recognition threshold (SRT) and word recognition score (WRS) values were similar between the two aided conditions.

Fig 2B shows consonant perception in quiet and noisy backgrounds. There were significant differences in consonant perception depending on the listening condition (*χ*^*2*^(2) = 18.175, *p* < 0.001 in a quiet condition; *χ*^*2*^(2) = 16.231, *p* < 0.001 in a steady noise condition; and *χ*^*2*^(2) = 23.792, *p* < 0.001 in a modulated noise condition). Post-hoc analysis with Wilcoxon signed-rank test (a Bonferroni correction was applied to set a significance level of *p* < 0.017) determined that consonant perception in a quiet condition was significantly increased when tested in the CROS/BiCROS condition versus the unaided (Z = −3.694, *p* < 0.001) and BAHA (Z = −3.444, *p* = 0.001) conditions. However, consonant perception in either noise condition was significantly decreased when tested in the BAHA condition as compared with in the unaided condition (Z = −3.456, *p* = 0.001 in a steady noise condition and Z = −3.924, *p* < 0.001 in a modulated noise condition) and CROS/BiCROS condition (Z = −2.974, *p* = 0.003 in a steady noise condition and Z = −3.399, *p* = 0.001 in a modulated noise condition).

When comparing the pseudobinaural benefits of speech perception in noise, SNR thresholds of K-HINT in the three different noise conditions are presented in Fig 2C. The mean SNR threshold ± SD in the unaided condition was −0.63 dB ± 1.90 dB when speech and noise were directed from the front (F_S_F_N_), −6.90 dB ± 3.48 dB when speech was directed at the better ear and noise was directed at the poorer ear (NH_S_HL_N_), and 4.80 dB ± 2.10 dB when speech was directed to the poorer ear and noise was directed at the better ear (HL_S_NH_N_). Clearly, speech perception in the unaided condition was best in the NH_S_HL_N_ condition and worst in the HL_S_NH_N_ condition [F(2, 40) = 222.412, *p* < 0.001 in a repeated-measures ANOVA, all p < 0.001 in post-hoc tests using the Bonferroni correction]. Speech perception with BAHA was of a similar pattern to in the unaided condition in the three different noise conditions [F(2, 40) = 95.331, *p* < 0.001 in a repeated-measures ANOVA with a Greenhouse–Geisser correction, all p < 0.001 in post-hoc tests using the Bonferroni correction]. The mean SNR threshold ± SD with BAHA was −1.53 dB ± 1.49 dB in the F_S_F_N_ condition, −7.27 dB ± 3.23 dB in the NH_S_HL_N_ condition, and 2.62 dB ± 2.52 dB in the HL_S_NH_N_ condition. However, speech perception with CROS/BiCROS was not different among any of the noise conditions [F(2, 40) = 1.345, *p* = 0.272 in a repeated-measures ANOVA with a Greenhouse–Geisser correction]. The mean SNR threshold ± SD with CROS was −1.52 dB ± 1.14 dB in the F_S_F_N_ condition, −3.19 dB ± 2.97 dB in the NH_S_HL_N_ condition, and −1.67 dB ± 4.91 dB in the HL_S_NH_N_ condition.

A repeated-measures ANOVA with a Greenhouse–Geisser correction determined that speech perceptions in the three different noise conditions differed significantly between listening conditions [F(2, 40) = 3.729, *p* = 0.046 for the F_S_F_N_ condition; F(2, 40) = 13.421, *p* < 0.001 for the NH_S_HL_N_ condition; and F(2, 40) = 32.683, *p* < 0.001 for the F_S_F_N_ condition]. For the F_S_F_N_ condition (summation), post-hoc tests using the Bonferroni correction revealed that speech perception in the CROS/BiCROS condition was significantly improved as compared with in the unaided condition (*p* = 0.028). Furthermore, speech perception in the BAHA condition elicited a slight improvement versus in the unaided and CROS/BiCROS conditions, though not in a statistically significant manner (*p* = 0.206 and *p* = 1.000, respectively). For the NH_S_HL_N_ condition (Squelch), post-hoc tests using the Bonferroni correction revealed that speech perception in the CROS/BiCROS condition was significantly decreased as compared with in the unaided (*p* = 0.006) and BAHA (*p <* 0.001) conditions. In other words, CROS/BiCROS delivered noise from the poorer ear to the better ear and could not suppress the delivered noise (no pseudobinaural squelch effect). However, speech perception in BAHA showed a slight improvement as compared with in the unaided condition, although not in a statistically significant way (*p* = 1.000). For the HL_S_NH_N_ condition (head shadow), post-hoc tests using the Bonferroni correction showed that both CROS/BiCROS and BAHA led to significantly better speech perception than did the unaided condition (*p* < 0.001 and p = 0.03, respectively). Additionally, CROS/BiCROS performed significantly better in reducing the head shadow effect than did BAHA (*p <* 0.001).

### Psychoacoustic performances

**Fig 3:**
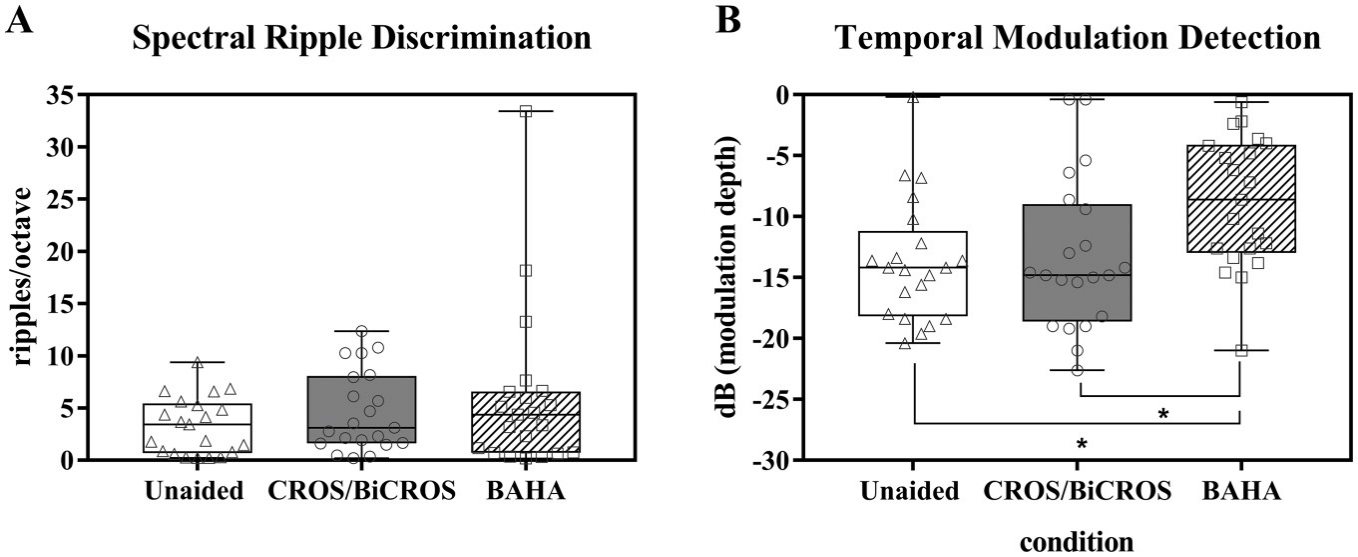
Psychoacoustic performance. SRD thresholds (A) and TMD thresholds (B) are shown for the three listening conditions. For the SRD test, a higher detection threshold indicates better SMD performance. For the TMD test, a more negative detection threshold implies better TMD performance. An asterisk (*) indicates a significant difference between two groups in post-hoc analysis (*p*-value was 0.05/3 based on Bonferroni correction).

Psychoacoustic performance for each listening condition is shown in Fig 3. SRD thresholds are shown in Fig 3A. For the SRD test, higher detection thresholds indicate better spectral resolution. There were no significant differences in SRD performance among the three listening conditions (*χ*^*2*^(2) = 3.349, *p* = 0.187). TMD thresholds at 10 Hz are shown in Fig 3B. Here, more negative detection thresholds imply better temporal resolution. There was a statistically significant difference in temporal resolution depending on listening conditions (*χ*^*2*^(2) = 14.095, *p* = 0.001). Post-hoc analysis with Wilcoxon signed-rank tests (a Bonferroni correction was applied to establish a significance level of *p* < 0.017) revealed that temporal resolution in the BAHA condition was significantly decreased as compared with in the unaided condition (Z = 3.720, *p* < 0.001) and the CROS/BiCROS condition (Z = 3.217, *p* = 0.001).

### Questionnaires

**Fig 4:**
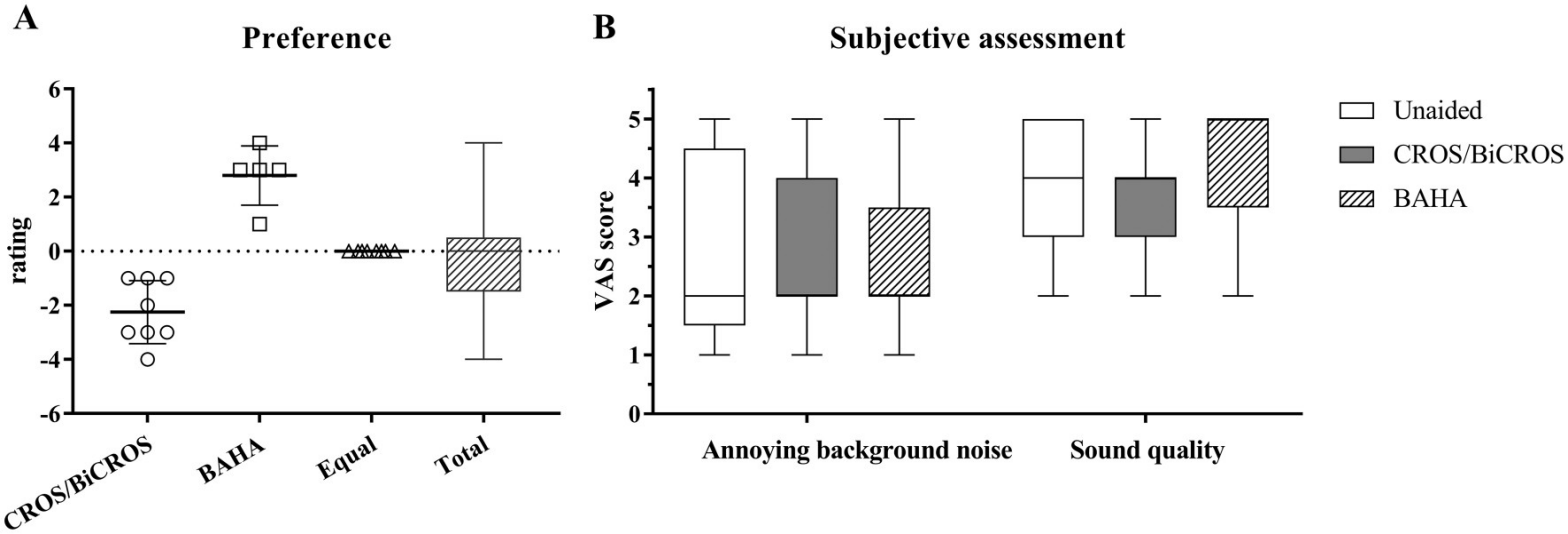
Subjective assessments. Panel A shows the study participant preferences for wireless CROS/BiCROS and soft-band BAHA. Panel B shows a six-item VAS for assessing background noise and sound quality in the three listening conditions. A higher score indicates that the sound was perceived better.

Hearing aid preference and subjective assessments are shown in Fig 4. Eight participants preferred a CROS/BiCROS device and five participants preferred a BAHA. The rest had no device preference. Mean rating score was 0.2. Annoying background noise was similar between listening conditions (*χ*^*2*^(2) = 0.353, *p* = 0.838), while speech quality significantly differed depending on listening condition (*χ*^*2*^(2) = 8.291, *p* = 0.016). However, there were no significant differences between listening conditions in post-hoc analysis with Wilcoxon signed-rank tests (Z = −1.213, *p* = 0.225 in unaided vs. CROS/BiCROS; Z = −0.994, *p* = 0.320 in unaided vs. BAHA; and Z = −1.869, *p* = 0.062 in CROS/BiCROS vs. BAHA).

### Factors associated with hearing aid benefit

To provide a customized rehabilitation strategy, a Pearson’s correlation was run to assess the relationship between device benefits (CROS/BiCROS or BAHA) and personal factors such as age, hearing threshold of the better ear, unaided TMD performance, and unaided SRD performance (Table 3). Hearing aid benefit was calculated as the difference of performance between aided and unaided conditions (device benefit = aided condition − unaided condition) for localization and speech recognition. The hearing thresholds averaged pure-tone thresholds at 0.5, 1, 2, and 4 kHz. The RMSEs of participants in the CROS/BiCROS condition were significantly increased in those who had a better hearing threshold in the better ear, better temporal resolution, and better spectral resolution (|*r*| = 0.441–0.518, all *p* < 0.05). On the contrary, that in the BAHA condition were significantly increased in participants who had a worse hearing threshold in the better ear, impaired temporal resolution, and impaired spectral resolution (|*r*| = 0.482–0.579, all *p* < 0.05). The benefit of a device that improves speech perception was significantly correlated with personal factors only in the CROS/BiCROS condition. The benefit of CROS/BiCROS for consonant perception in a quiet environment was significantly increased in participants who had worse hearing thresholds in the better ear, impaired spectral resolution, and impaired temporal resolution (|*r*| = 0.516–0.589, all *p* < 0.05). The benefit of CROS/BiCROS for consonant perception in a steady noise was also significantly increased in participants who had impaired temporal resolution (*r* = 0.490, *p* = 0.24). There was no significant correlation between personal factors and benefit of a device that improved speech-in-noise perception by summation effect (all *p* > 0.05). The benefit of CROS/BiCROS for speech-in-noise perception was significantly increased with worse spectral resolution (*r* = 0.558, *p* = 0.009) when speech came from the direction of the better ear and noise came from the direction of the poorer ear (NH_S_HL_N_, squelch effect). However, it was significantly increased with younger age (*r* = 0.569, *p* = 0.00) and better spectral resolution (*r* = −.489, *p* = 0.025) when speech and noise came directionally from the poorer ear (HL_S_NL_N_, head shadow).

**Table 3:** Correlation coefficients of hearing aid benefits on localization and speech perception.

Variable			Age (years)	Pure-tone threshold in better ear (dB HL)	Unaided SRD (rpo)	Unaided TMD (dB)
Localization (negative value of device benefit indicates positive benefit from wearing a device)						
RMSE	CROS benefit	*R*	−0.391	−0.518*	0.441*	−0.516*
		*P*	0.080	0.016	0.045	0.017
	BAHA benefit	*R*	0.368	0.579**	−0.515*	0.482*
		*P*	0.101	0.006	0.017	0.027
Consonant test (positive value of device benefit indicates positive benefit from wearing a device)						
In a quiet environment	CROS benefit	*R*	0.393	0.544*	−0.516*	0.589**
		*P*	0.078	0.011	0.017	0.005
	BAHA benefit	*R*	0.051	−0.155	−0.008	0.126
		*P*	0.827	0.502	0.971	0.585
In a steady noise environment	CROS benefit	*R*	0.126	−0.153	−0.107	0.490*
		*P*	0.586	0.509	0.645	0.024
	BAHA benefit	*R*	0.032	−0.198	−0.015	0.077
		*P*	0.891	0.390	0.947	0.739
In a modulated noise environment	CROS benefit	*R*	0.182	0.121	−0.379	0.312
		*P*	0.430	0.601	0.091	0.168
	BAHA benefit	*R*	0.133	0.030	−0.384	0.278
		*P*	0.565	0.898	0.086	0.222
K-HINT (negative value of device benefit indicates positive benefit from wearing a device)						
Summation	CROS benefit	*R*	−0.184	−0.314	−0.066	0.014
		*P*	0.424	0.166	0.777	0.951
	BAHA benefit	*R*	0.029	−0.149	−0.116	0.076
		*P*	0.902	0.520	0.618	0.744
Squelch	CROS benefit	*R*	−0.425	−0.300	0.558**	−0.352
		*P*	0.055	0.187	0.009	0.118
	BAHA benefit	*R*	0.088	0.130	0.170	−0.095
		*P*	0.705	0.573	0.461	0.683
Head shadow	CROS benefit	*R*	0.569**	0.200	−0.489*	0.429
		*P*	0.007	0.384	0.025	0.052
	BAHA benefit	*R*	−0.060	−0.268	−0.028	0.129
		*P*	0.797	0.241	0.904	0.577

Asterisks indicate a statistically significant difference (* = *p* < 0.05, ** = *p* < 0.01).

## Discussion

This study provides possible counseling information to be considered when choosing a noninvasive rehabilitative option in individuals with severe to profound unilateral SNHL. Wireless CROS/BiCROS hearing aids and soft-band BAHAs had no benefit for improving horizontal localization ability (Fig 1). In fact, wireless CROS/BiCROS hearing aids had a negative effect on localization ability. Notably, when wearing a CROS/BiCROS hearing aid, localization was significantly decreased in participants who had better hearing thresholds in the better ear, better spectral resolution, or better temporal resolution (Table 3). Sound localization in the horizontal plane depends on the ability to detect differences in arrival time (interaural time differences) and sound level (interaural level differences). Some studies have reported that monaural listeners can localize in the horizontal plane by using either spectral cues [1] or perceived level differences [29] in the signal as it moves from the impaired ear to the better hearing ear. However, a CROS/BiCROS hearing aid seemed to have disrupted the monaural level and spectral cues for localization in the horizontal plane [30]. A soft-band BAHA did not significantly improve or decrease the localization ability. In this study, the benefit of BAHA for localization was significantly decreased in participants who had worse hearing thresholds in the better ear, impaired spectral resolution, or impaired temporal resolution (Table 3). The BAHA for unilateral SNHL was originally used only in participants who had normal hearing on the contralateral side, as indicated by a pure-tone average air-conduction hearing threshold (measured at 0.5, 1, 2, and 3 kHz) of better than or equal to 20 dB HL. Thus, a paired t-test was conducted on a sample of 12 participants who had normal hearing on the contralateral side (pure-tone average hearing threshold ≥ 20 dB HL) to determine whether there was a statistically significant mean difference of RMSE between the unaided and BAHA conditions. However, wearing a BAHA caused a slight increase of mean RMSE in comparison with in the case of the unaided condition (mean RMSEs were 42.2° for the unaided condition and 46.7° for the BAHA condition), which was not a statistically significant finding (*t*(11) = −1.239, *p* = 0.241).

A CROS/BiCROS hearing aid significantly enhanced consonant perception in a quiet situation as compared with in the unaided and BAHA conditions (Fig 2B), and this benefit was significantly increased in participants who had worse hearing thresholds in the better ear, impaired spectral resolution, and impaired temporal resolution (Table 3). Separately, BAHA significantly reduced consonant perception versus in the unaided condition when noise was presented from front, regardless of background noise (Fig 2B). This result might be due to the transcranial attenuation of delivered sound or the use of an ear plug in the better ear. Both devices (CROS/BiCROS hearing aid and BAHA) evaluated in this study significantly improved speech-in-noise perception by reducing the head shadow effect (Fig 2C). However, the CROS/BiCROS hearing aid was more effective in attenuating the head shadow effect than the BAHA, although the CROS/BiCROS hearing aid significantly reduced speech-in noise perception by way of the squelch effect as compared with the unaided condition. The BAHA did not have a negative effect on speech-in-noise perception when noise was presented to the poorer ear. Previous studies have suggested that unaided conditions are better than wearing a CROS hearing aid or BAHA when noise originates from the poorer ear side, since both devices transmit the noise to the better ear [6, 14]. Similar to our results, other researchers demonstrated a greater disadvantage with the CROS device versus the BAHA when noise was transmitted to the poorer ear. While a CROS/BiCROS hearing aid can help to attenuate the head shadow effect more effectively than a BAHA, the noise transmitted to the better ear in the former interferes with speech perception more significantly than in the case of the BAHA. When unilateral SNHL patients were younger or had better spectral resolution in their better ear, CROS/BiCROS was found to transmit speech and noise more successfully (Table 3).

When we compared the subjective discomfort in the noisy background and sound quality, participants rated both hearing devices similarly to the unaided condition (Fig 4B). The preference results also showed equal ratings for the CROS/BiCROS hearing aid and the BAHA (Fig 4B). It is important to assess participant self-perceived satisfaction with these devices because tests performed in an audio booth are generally not representative of everyday listening situations.

This study has several limitations. First, this study had no trial period for participants to adjust to the hearing devices. Psychoacoustic results showed that temporal resolution decreased when sound was delivered from the poorer ear to the better ear through a BAHA (Fig 3). In other words, using a soft-band BAHA caused a difference in arrival time as the sound was delivered from the impaired ear to the better ear. This difference could help to improve the localization ability in the horizontal plane. Thus, the long-term benefits of both hearing devices need to be assessed. Second, some participants in this study had mild hearing loss in the contralateral ear. However, subgroup analysis with a sample of 12 participants who had normal hearing in the contralateral side did not differ in terms of localization and speech perception (S1 Table). Third, this study used the Baha 3 Power, but the Baha 5 Power was recently made available with improved signal processing. Fourth, clinical information such as the duration of unilateral SNHL or whether the study participants have previously used conventional hearing aids was lacking.

## Conclusion

In summary, sound localization ability in the horizontal plane was significantly decreased in the CROS/BiCROS condition because it diminished the monaural level and spectral cues for monaural listeners. A CROS/BiCROS hearing aid provided a slight but significant additional benefit for speech perception in a quiet and speech-in-noise perception when speech and noise were presented from the front. Both the CROS/BiCROS hearing aid and BAHA helped reduce the head shadow effect, and they significantly enhanced speech-in-noise perception when the target speech was presented to the poorer ear. A CROS/BiCROS hearing aid was superior to a BAHA in overcoming the head shadow effect, but it appeared to have a rather negative effect when the noise was delivered to the better ear. It should be noted that the performance of the CROS/BiCROS hearing aid was significantly influenced by age, hearing thresholds in the better ear, and unaided psychoacoustic performances and these factors should be considered when counseling patients in hearing aid clinics. Despite the lack of device acclimatization, we believe that this study provides potential counseling information for use in hearing aid clinics for the management of patients with severe to profound unilateral SNHL.

## Supporting information

S1 Table Subgroup analysis with a sample of 12 participants who had normal hearing on the contralateral side. Normal hearing on the contralateral side is defined as pure-tone average air-conduction hearing threshold (measured at 0.5, 1, 2, and 3 kHz) of better than or equal to 20 dB HL. p* for unaided and CROS/BiCROS conditions, *p*^*¶*^ for unaided and BAHA conditions, *p*^*†*^ for CROS/BiCROS and BAHA conditions.
(DOCX)
