# Supplementary material for: A comparison between wireless CROS/BiCROS and soft-band BAHA for patients with unilateral hearing loss
Source: PLoS One. 2019 Feb 21;14(2):e0212503. doi: 10.1371/journal.pone.0212503 (PMC6383877; doi:10.1371/journal.pone.0212503)
Supplement: S1 Table — Normal hearing on the contralateral side is defined as pure-tone average air-conduction hearing threshold (measured at 0.5, 1, 2, and 3 kHz) of better than or equal to 20 dB HL. p* for unaided and CROS/BiCROS conditions, p¶ for unaided and BAHA conditions, p† for CROS/BiCROS and BAHA conditions. (DOCX) [file pone.0212503.s001.docx]

**S1 Table.** Subgroup analysis with a sample of 12 participants who had normal hearing on the contralateral side.

| Variable | | Mean ± standard deviation | | | RM-ANOVA | | | Post-hoc tests | | |
| --- | --- | --- | --- | --- | --- | --- | --- | --- | --- | --- |
|  |  | Unaided | CROS/BiCROS | BAHA | *df* | *F* | *p* | *p^*^* | *p^¶^* | *p^†^* |
| RMSE | | 42.2 ± 15.7 | 66.1 ± 17.2 | 46.7 ± 17.4 | 2 | 10.155 | **0.001** | **0.006** | 0.724 | **0.048** |
| Consonant | Quiet | 91.2 ± 4.0 | 94.4 ± 3.9 | 86.4 ± 15.2 | 1.144 | 4.43 | 0.052 | **0.023** | 0.502 | 0.095 |
|  | steady noise | 93.4 ± 5.0 | 93.2 ± 6.0 | 83.5 ± 15.2 | 2 | 4.183 | **0.046** | 1.000 | 0.178 | 0.127 |
|  | modulated noise | 95.1 ± 3.1 | 92.7 ± 4.5 | 86.4 ± 11.3 | 1.089 | 8.432 | **0.012** | 0.274 | **0.029** | 0.060 |
| HINT | Summation | −1.1 ± 1.5 | −1.8 ± 1.0 | −1.7 ± 1.4 | 2 | 1.267 | 0.301 | 0.290 | 0.849 | 1.000 |
|  | Squelch | −8.0 ± 2.2 | −3.1 ± 3.0 | −8.7 ± 1.9 | 1.348 | 21.574 | **<.001** | **0.002** | 0.674 | **0.001** |
|  | Head shadow | 4.1 ± 1.7 | −3.5 ± 3.6 | 2.3 ± 2.1 | 2 | 35.090 | **<.001** | **<.001** | **0.023** | **0.001** |

Normal hearing on the contralateral side is defined as pure-tone average air-conduction hearing threshold (measured at 0.5, 1, 2, and 3 kHz) of better than or equal to 20 dB HL.

*p^*^* for unaided and CROS/BiCROS conditions, *p^¶^* for unaided and BAHA conditions, *p^†^* for CROS/BiCROS and BAHA conditions
